# The impact of T cell repertoire on mortality: Findings from the Health and Retirement Study

**DOI:** 10.1093/geroni/igaf122.2968

**Published:** 2025-12-31

**Authors:** Zach Flaten, Ryan Martinez, Sithara Vivek, Shijia Zhu, Jessica Faul, Eileen Crimmins, Gokul Seshadri, Bharat Thyagarajan

**Affiliations:** University of Minnesota, Minneapolis, Minnesota, United States; University of Minnesota, Minneapolis, Minnesota, United States; University of Minnesota, Minneapolis, Minnesota, United States; University of Minnesota, Minneapolis, Minnesota, United States; University of Michigan, Ann Arbor, Michigan, United States; University of Southern California, Los Angeles, California, United States; University of Minnesota, Minneapolis, Minnesota, United States; University of Minnesota, Minneapolis, Minnesota, United States

## Abstract

The T and B cell repertoire is the sum of all the T-cell receptors (TCRs) and B cell receptors (BCRs) in an individual and is an indicator of the cumulative exposure of immune challenges over the life course. The biological importance of T/B cell repertoire diversity in a general population of older adults remains unclear. In the Health and Retirement Study, we used MiXCR on bulk RNAseq data to estimate T/B receptor chain numbers (total clones) and T/B receptor chain diversity (number of unique clones) for 3,758 participants. We evaluated the demographic (age, sex, race) and other factors (smoking, CMV seroprevalence) associated with T/B cell repertoire using type-II ANOVA. We used unconditional logistic regression models to evaluate the association between T/B cell repertoire and 4-year mortality. All the models were adjusted for age, sex, race, CMV seropositivity, smoking status, total number of sequencing reads, and total T cell/ B cell counts. The number and diversity of T and B cell receptor chains identified were higher among African Americans as compared to other racial groups. The number and diversity of TCR chains were more common in women, elevated in CMV seropositive individuals. TCR chains were more common in never smokers, but the TCR diversity was higher in current smokers. Moreover, higher numbers of TCR chains (OR: TRA=0.74;p=0.0008, TRB=0.76;p=0.0018) and TCR clonal diversity (OR: TRA=0.66; p < 0.0001, TRB=0.7; p < 0.0001) were significantly associated with reduced mortality. Further research is needed to understand mechanisms by which TCR impact population health.

